# Amelioration of Acute Sequelae of Blast Induced Mild Traumatic Brain Injury by N-Acetyl Cysteine: A Double-Blind, Placebo Controlled Study

**DOI:** 10.1371/journal.pone.0054163

**Published:** 2013-01-23

**Authors:** Michael E. Hoffer, Carey Balaban, Martin D. Slade, Jack W. Tsao, Barry Hoffer

**Affiliations:** 1 Spatial Orientation Center, Department of Otolaryngology, Naval Medical Center San Diego, San Diego, California, United States of America; 2 Departments of Otolaryngology, Neurobiology, Communication Sciences and Disorders, and Bioengineering, University of Pittsburgh, Pittsburgh, Pennsylvania, United States of America; 3 Department of Internal Medicine, Yale University, New Haven, Connecticut, United States of America; 4 Wounded, Ill and Injured Directorate (M9), US Navy Bureau of Medicine and Surgery, Washington, D.C., United States of America; 5 Department of Neurosurgery, Case Western University, Cleveland, Ohio, United States of America; University of Toronto, Canada

## Abstract

**Background:**

Mild traumatic brain injury (mTBI) secondary to blast exposure is the most common battlefield injury in Southwest Asia. There has been little prospective work in the combat setting to test the efficacy of new countermeasures. The goal of this study was to compare the efficacy of N-acetyl cysteine (NAC) versus placebo on the symptoms associated with blast exposure mTBI in a combat setting.

**Methods:**

This study was a randomized double blind, placebo-controlled study that was conducted on active duty service members at a forward deployed field hospital in Iraq. All symptomatic U.S. service members who were exposed to significant ordnance blast and who met the criteria for mTBI were offered participation in the study and 81 individuals agreed to participate. Individuals underwent a baseline evaluation and then were randomly assigned to receive either N-acetyl cysteine (NAC) or placebo for seven days. Each subject was re-evaluated at 3 and 7 days. Outcome measures were the presence of the following sequelae of mTBI: dizziness, hearing loss, headache, memory loss, sleep disturbances, and neurocognitive dysfunction. The resolution of these symptoms seven days after the blast exposure was the main outcome measure in this study. Logistic regression on the outcome of ‘no day 7 symptoms’ indicated that NAC treatment was significantly better than placebo (OR = 3.6, p = 0.006). Secondary analysis revealed subjects receiving NAC within 24 hours of blast had an 86% chance of symptom resolution with no reported side effects versus 42% for those seen early who received placebo.

**Conclusion:**

This study, conducted in an active theatre of war, demonstrates that NAC, a safe pharmaceutical countermeasure, has beneficial effects on the severity and resolution of sequelae of blast induced mTBI. This is the first demonstration of an effective short term countermeasure for mTBI. Further work on long term outcomes and the potential use of NAC in civilian mTBI is warranted.

**Trial Registration:**

ClinicalTrials.gov NCT00822263

## Introduction

Mild traumatic brain injury (mTBI) is the most common injury seen in the current conflicts in Iraq and Afghanistan and an increasingly common injury in modern society. An estimated 19.5–22.8% of all returning deployed troops suffer from mTBI [1]. The most common cause of mTBI is blast exposure from improvised explosive devices (IEDs) or other explosive ordinances. Over the last three decades the mechanisms, characteristics, diagnostic schemes, and treatment strategies for blunt head trauma have been studied extensively. Unfortunately, many of the lessons learned from blunt head trauma cannot be applied automatically to blast injured subjects [2].

Disentangling the neurological features of mTBI from PTSD is important for improving diagnosis and treatment of low-level blast injuries [3]. Many previous studies were conducted well after the actual blast injury and focused on the role of mTBI as a precursor of PTSD [1], [4]. Diagnosis of mTBI requires a documented traumatic event, with a transient loss or alteration of consciousness, accompanied by at least one of a list of neurologic, neurotologic, or cognitive symptoms [5]. One highly prevalent symptom is dizziness, a subjective marker of balance dysfunction.

N-acetylcysteine (NAC) is a logical choice for a field-based clinical trial. NAC is the active agent in Mucomyst, an FDA approved medication with a forty-year safety history. NAC is an effective neuroprotective agent in animal ischemia-reperfusion cerebral stroke models [6], [7], [8], a rodent closed head trauma model [9], a sensory nerve axotomy model [10] and inner ear neuronal death after noise exposure [11], [12]. Although these neuroprotective effects of NAC appear to be mediated by both antioxidant and anti-inflammatory effects [7], [8], [13], [14], [15], NAC also acts indirectly at metabotropic glutamate receptors to counteract cocaine-induced disruption of nucleus accumbens [16]. Further, oral NAC and placebo preparations have been utilized on over 1000 Marines and Sailors in on-going approved clinical investigations to test protection from noise-induced hearing loss. Finally, NAC has limited capability to cross the normal blood-brain barrier [13], which suggests that it can preferentially enter tissue at sites of post-traumatic barrier disruption.

This protocol administered a loading dose of NAC to exposed U.S. Service members within 0–72 hours after blast exposure and followed a set of subjective and objective outcome measures of mTBI. The addition of NAC to standard treatment produced a much higher rate of symptom resolution than placebo at seven treatment days.

## Materials and Methods

### Overview

**Figure 1:**
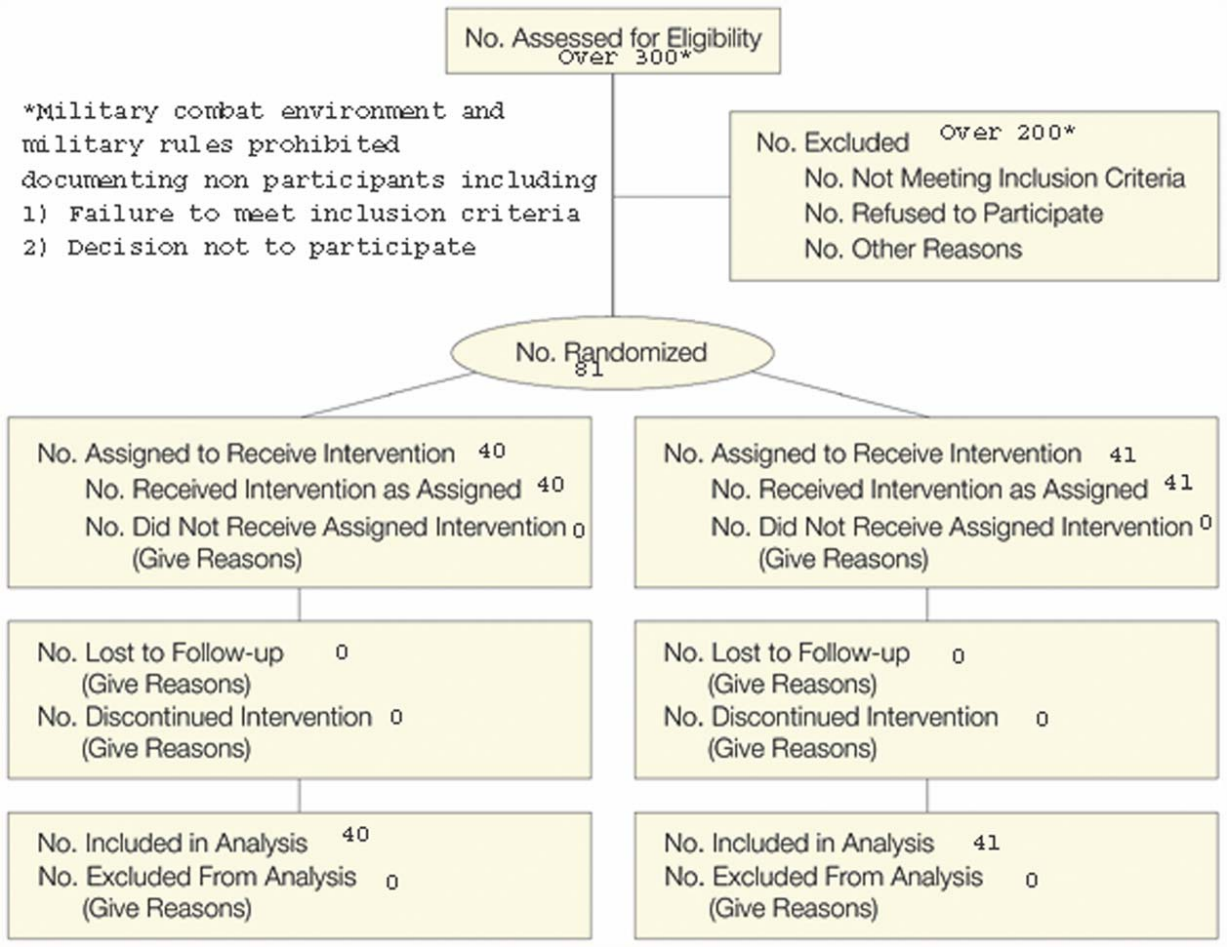
Consort Flow Diagram.

The study is the first double blind, placebo controlled study examining mTBI to take place during active combat. It was subject to design constraints because the participants were active duty personnel who never departed from an active combat zone. Diagnosis and treatment of mTBI were delivered by medical personnel in a medical facility within that active combat zone. Operational manpower needs and military policy precluded follow-up beyond the initial seven day treatment period. Enrollment in the study ended with the conclusion of combat operations in Western Iraq. A flow diagram for the study is shown in Figure 1.

### Ethics

The protocol was approved by the Joint Theatre Trauma and Research Office in Baghdad, Iraq and the theatre Institutional Review Board (IRB) at Brooke Army Medical Center.

### Detailed Methods

The protocol for this trial and supporting CONSORT checklist are available as supporting information; see Checklist S1 and Protocol S1. The study was conducted at Al Taqaddum Level II Medical Facility (TQ) in Iraq. US Military Service members exposed to a significant blast in the Al Anbar province of Iraq were evaluated at TQ as rapidly as feasible (given operational and weather concerns). The October 2007 Joint Service Surgeon General and DoD Health Affairs criteria were utilized to diagnose mTBI [5]. Briefly, this definition specifies that there is an exposure, a brief alteration of consciousness, and then manifestation of any one of a number of mTBI symptoms. Exposure was defined as feeling a blast wave or being in a vehicle that was damaged by a blast wave. Alteration of consciousness required a report by another individual on the scene. Balance dysfunction, confusion, headache, sensorineural hearing loss, impaired memory and sleep disturbances were considered as mTBI symptoms. Individuals with moderate to severe TBI, significant orthopedic injuries, or those requiring computerized tomography scans, evacuation from theatre, or emergent surgeries were excluded from this study.

All subjects underwent a comprehensive history and physical exam by the Principal Investigator (PI). A finding of balance dysfunction required subjective dizziness, an abnormal head thrust test (abnormal eye motion after quick turning of the subjects head by the investigator [17]), an abnormal Romberg/tandem Romberg test (excessive swaying or falling while attempting to stand still with eyes closed and arms extended [18]), and an abnormal dynamic gait index (DGI) score (a dedicated set of walking tasks scored by the PI [19]) of <22. Criteria for resolution of balance dysfunction were no subjective dizziness, normal head thrust and Romberg tests, and a normal DGI. Audiometric testing identified hearing loss and its resolution. Confusion, headache, sleep disturbances, and impaired memory were based upon self-reports; resolution of these symptoms required a report that “I feel as good as before the blast.” Sleep disorders were difficult to assess because many subjects were sleep deprived upon arrival; they were diagnosed when clinical judgment indicated that initial exhaustion was eliminated.

All subjects diagnosed with mTBI were given standard treatment for signs and symptoms as needed, which was identical to treatment administered for mTBI in US military and civilian hospitals. Treatment included an individualized exercise program, symptomatic headache medications (non-narcotic medicine such as topiramate, sumatriptin, and ibuprofen to treat associated symptoms), low level of activity with proper rest, and controlled mental stimulation. All subjects were also informed about the study and offered an opportunity to participate. All individuals were advised that participation was voluntary and would have no impact on their ability to receive care or on their ability to receive standard of care for their injury. All individuals who desired to consider participating were given an informed consent form to read and allowed ample opportunity to ask questions. Those agreeing to participate completed the IRB approved informed consent document. All participants received a pre-randomized bottle containing 500 mg tablets of either NAC or placebo, prepared by the pharmacy of a major United States Military Medical Center. Assignment was based solely on order of accession into the study. Simple randomization was utilized to create an assignment list (randomization schedule) for subjects to be assigned to either the Placebo or NAC group as no interim analyses were to be conducted and this method assures that each treatment assignment is completely unpredictable [20]. The randomization schedule was created prior to subject accrual and resulted in a 49·4% to 50·6% split in subjects between the two groups. Participants, providers, and evaluators were all blinded to which treatment group each subject was assigned.

The 4 gram loading dose was witnessed and then bottles were name labeled and given to the subject's corpsman, medic or a nurse (for subjects remaining at TQ). Beginning 18–24 hours after the loading dose, subjects were given 4 grams daily (in two divided doses of 2 gm morning and night) for 4 days. The dose was then reduced to 3 gm daily [in two divided doses of 1.5 gm morning and night]. Every dose was witnessed by a corpsman, medic, nurse, or doctor.

The Controlled Oral Word Association Test (COWA) [21], animal naming (AN), and timed Trail Making Tests (TMT) A and B [22], [23], [24], [25] were administered as neuropsychological tests of executive function. Normative TMT data were estimated from published age-normed data [26], assuming an equal mixture of 18–24 and 25–34 year old groups. The initial clinical assessment, history, physical, DGI, hearing, and neuropsychology tests were repeated at three and seven days after enrollment.

The study was designed originally to examine the effects of NAC versus placebo in subjects also receiving standard treatment (measured exercise program and non-narcotic headache medicines). Because availability of transportation in the combat zone affected a patient's arrival time at TQ, we also examined secondarily the impact of early (within 24 hours) versus delayed (26–72 hours after injury) diagnosis and treatment on outcomes. Time of arrival at TQ was based purely on distance of the injury from TQ and the safety and availability of transportation back to TQ. While it is impossible to control for all confounders between the early and late group, it should be noted that individuals in both groups came from the same sets of combat units, were doing the same duty, and were living under the same conditions. The primary endpoint was the percentage of subjects free of all symptoms of mild TBI on D7 where D7 was defined 7 days since the loading dose of medication was given. It was assumed that 80% of subjects in the placebo arm of the study would still have at least one symptom on day 7. The study was sized to determine if NAC was able to reduce that 80% to 50%, hence only 50% of subjects would still have a symptom of mild TBI on day 7, with a 95% level of confidence, 80% power, and a 1∶1 ratio of control to experimental subjects. Based upon these criterion, 38 subjects were required in each arm of the study [27]. Secondary endpoints were balance dysfunction resolution, absence of headache, confusion, memory problems, abnormal sleep, trail making A, trail making B, controlled oral word association (COWA), and animal naming (AN) all on day 7, as well as the percentage of subjects free of all symptoms of mild TBI three days post initial treatment.

Student t-tests were utilized to determine differences between groups for age, distance from blast and number of blast exposures at time of assignment for treatment. Pooled variance determined significance unless group variances were significantly different, in which case Satterthwaite's method was used. MLR and Least Significant Differences (LSD) tests were used for multiple comparisons. Fisher's exact test was used to test group differences for categorical variables of sex and job title.

For the primary analysis, unadjusted logistic regression was used to model the effect of NAC on resolution of symptoms D7. For secondary analyses, multivariate logistic regression modeled the effects of NAC, early treatment, number of previous blast occurrences, age, distance from blast, as well as all two-way interactions, on resolution of symptoms on D7. Parsimonious models [28] were constructed utilizing a backward elimination strategy with a significance level to stay of α = 0·05. In a similar manner, multivariate linear regression modeled predictors of the number of D7 symptoms. Parallel analyses of day 3 data revealed no significant effects.

## Results

### General subject characteristics

Eighty-one subjects participated in the study (eighty males and one female). The age range was 18–43 years of age with a median age of 22 years. The subject demographic data (Table 1) did not differ between the NAC and the placebo groups, between subjects treated early (≤24 hours) or late (26–72 hours) after injury, or between any of the four treatment groups defined by combinations of these factors. The groups did not differ with respect to other medicines being utilized. Neither alcohol nor drug abuse were factors because the study was conducted in a controlled combat setting. Theatre guidelines prohibited a detailed documentation or tracking of those who declined participation but over 1/3 of those seen declined to participate in the trial. No participant suffered unintended effects, side effects, or harm. In particular, no GI upset was reported.

**Table 1:** Study Group Characteristics.

Continuous Covariates	Treatment Before 24 Hours				Treatment After (26–72 Hours)			
	Group A: -NAC Control(n = 31)		Group B: +NAC (n = 29)		Group C: -NAC Control (n = 9)		Group D: +NAC (n = 12)	
	Mean	Std Dev	Mean	Std Dev	Mean	Std Dev	Mean	Std Dev
Age	23.58	4.16	24.92	6.52	25.42	6.19	27.68	6.96
Distance from blast (feet)†	17.42	7.40	16.79	5.18	14.67	2.69	15.83	4.69
Number of Blast Exposures (inclusive)	2.23	3.60	1.45	1.21	1.89	1.69	6.58	8.31‡

† include vehicle dimensions (74/81 participants) and IED distance.

‡ Greater variance reflects the random inclusion of the two oldest individuals in the study population.

All subjects in the study had objective clinical evidence of balance dysfunction. Table 2 summarizes the distribution of additional mTBI symptoms. Only one subject had an isolated finding of balance dysfunction. Hearing loss, headache and confusion were the next most prevalent features.

**Table 2:** Distribution of mTBI Symptoms Co-morbid with Balance Dysfunction: Entry to Study.

*Number of Symptoms*	*Hearing loss*	*Headache*	*Confusion*	*Memory Problem*	*Sleep Abnormal*	*Number of Subjects*
*1*	*−*	*−*	*−*	*−*	*−*	*1*
2	+	−	−	−	−	6
	−	+	−	−	−	6
	−	+	−	−	−	6
*3*	*−*	*+*	*−*	*+*	*−*	*7*
	*−*	*+*	*+*	*−*	*−*	*6*
	*+*	*−*	*−*	*−*	*+*	*1*
	*+*	*+*	*−*	*−*	*−*	*8*
	*+*	*−*	*−*	*+*	*−*	*2*
	*+*	*−*	*+*	*−*	*−*	*13*
4	+	−	+	+	−	1
	+	+	+	−	−	17
	+	+	−	+	−	7
	+	+	−	−	+	1
*5*	*+*	*+*	*+*	*−*	*+*	*1*
Subjects Symptomatic	57	53	42	17	3	81

### Symptom emergence during the study

The prevalence of mTBI symptoms varied with the latency between blast exposure and examination. Hearing loss emerged on subsequent study days in 3 subjects, headache developed in 8 subjects, memory problems in 2 subjects and abnormal sleep in 10 subjects. Symptoms persisting at D7 are shown in Table 3.

**Table 3:** Unresolved mTBI Symptom Patterns on Treatment Day 7.

*Number of Symptoms*	*Balance dysfunction*	*Hearing loss*	*Headache*	*Confusion*	*Memory Problem*	*Sleep Abnormal*	*Number of Subjects*
1	*−*	*+*	*−*	*−*	*−*	*−*	*3*
	*−*	*−*	*+*	*−*	*−*	*−*	*4*
	*−*	*−*	*−*	*+*	*−*	*−*	*3*
	*+*	*−*	*−*	*−*	*−*	*−*	*4*
2	−	−	+	−	−	+	1
	−	+	+	−	−	−	2
	−	−	+	+	−	−	1
	+	−	−	−	−	+	3
	+	+	−	−	−	−	3
	+	−	+	−	−	−	6
3	*−*	*+*	*+*	*+*	*−*	*−*	*1*
	*+*	*+*	*+*	*−*	*−*	*−*	*3*
	*+*	*−*	*+*	*−*	*−*	*+*	*2*
	*+*	*−*	*+*	*+*	*−*	*−*	*2*
4	+	−	+	+	−	+	1
5	*+*	*+*	*+*	*−*	*+*	*+*	*1*
Subjects Symptomatic	25	13	24	8	1	8	40

### Treatment effects

**Figure 2:**
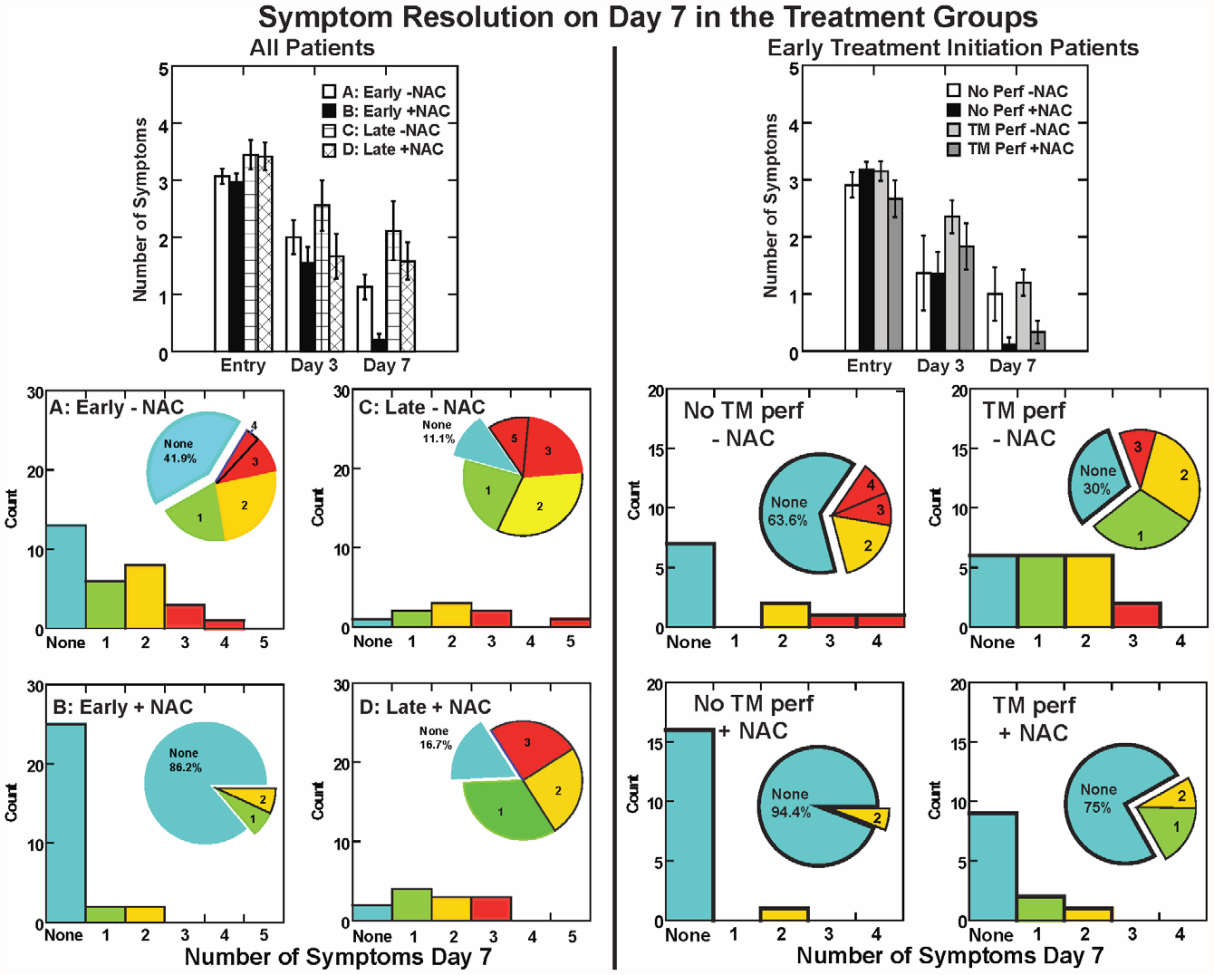
The number of symptoms for four groups of patients. The upper left panel shows results for all patients in the study. The upper right graph illustrates the impact of tympanic membrane perforations in the patients seen within 24 hours of blast exposure The distribution of the data on day 7 are shown for each group in the lower graphs, with a pie chart to indicate the percentage with no residual symptoms.

The NAC group was significantly more likely to have symptom resolution D7 (OR = 3.60, p = 0.0062, R^2^ = 0.37). In secondary analyses, both NAC treatment and early treatment initiation contributed independently to symptom resolution (Figure 2, left panels). There were independent main effects of both NAC treatment (F_1, 78_ = 10·28, p<0.01) and treatment initiation time (F_1, 78_ = 10·91, p<0.01) on the number of D7 symptoms (R^2^ = 0.21). The early treatment group given NAC had the fewest D7 symptoms (p<0·01, R^2^ = 0.24 re: each other group by LSD tests). The early treatment group without NAC showed fewer symptoms than the late treatment group without NAC (p<0.05). The late treatment group given NAC showed an intermediate number of symptoms that did not differ significantly from either early or late treatment groups without NAC There were no significant treatment group differences in the total number of symptoms upon entry (R^2^ = 0.03) or on treatment day 3 (R^2^ = 0.04).

Logistic regression on the outcome of ‘no D7 symptoms’ confirmed that NAC treatment was significantly better than placebo (p<0.005) and early intervention was significantly better than later intervention (p<0.005, max-rescaled R^2^ = 0.37)). The odds of complete symptom resolution for Group B (early treatment with NAC) were greater than the other groups (Tables 4 and 5, p<0.001). Parsimonious logistic regression models for balance dysfunction resolution and the absence of headache on day seven also showed significant effects for NAC and early treatment initiation (p<0.05), with no significant effects of covariates or interactions.

**Table 4:** Treatment Effects: Day 7 Symptoms.

			No Symptoms- Day 7			No Symptoms (Excluding Residual hearing loss-Day 7		
Timing of Treatment	Treatment	n	Odds Ratio*	Max Rescaled R2	Pr>ChiSq	Odds Ratio*	Max Rescaled R2	Pr>ChiSq
After 24 hours	Placebo	9	1 (reference)	0.38	0.0002	1 (reference)	0.34	0.0005
After 24 hours	NAC	12	1.60 (0.12, 20.99)			2.67 (0.23, 31.07)		
Before 24 hours	Placebo	31	5.05 (0.56, 45.64)			5.78 (0.64, 52.03)		
Before 24 hours	NAC	29	38.40 (3.88, 379.68)			38.40 (3.88, 379.68)		

* 95% Confidence Interval.

**Table 5:** Treatment Effects: Day 7 Symptoms.

			No Balance Dysfunction - Day 7			No Headache -Day 7		
Timing of Treatment	Treatment	n	Odds Ratio*	Max Rescaled R2	Pr>ChiSq	Odds Ratio*	Max Rescaled R2	Pr>ChiSq
After 24 hours	Placebo	9	1 (reference)	0.21	0.0185	1 (reference)	0.25	0.0117
After 24 hours	NAC	12	4.00 (0.64, 25.02)			2.80 (0.46, 16.93)		
Before 24 hours	Placebo	31	3.17 (0.66, 15.12)			3.64 (0.76, 17.46)		
Before 24 hours	NAC	29	17.33 (2.78, 108.06)			27.00 (3.67, 198.69)		

* 95% Confidence Interval.

Confusion resolved markedly after early treatment; 59/60 subjects treated early had no confusion D7, compared with only 14/21 subjects treated later (exact test, p<0.001). The parsimonious logistic regression model showed that D7 confusion was predicted by both the time of treatment (p<0.05) and the distance from the blast (p<0.05). No significant effects emerged in the parsimonious logistic regression models for hearing loss, memory problems and abnormal sleep. Despite the low prevalence of abnormal sleep in this series, it was significantly less likely (exact test, p = 0.029) in the NAC treated subject population on day seven (1/41) than in the placebo treated subjects (7/40).

### Neuropsychological treatment effects

Results on entry were significantly poorer than published normative Trail making Test A and B (TMTA and TMTB) data for subjects who were in the age ranges of 18–34 years [26] (Table 6, z-tests, p<0.001). Controlled oral word association (COWA) and animal naming (AN) were within normal limits.

**Table 6:** Neuropsychological Tests: TMT at Study Entry.

			Trail Making A (seconds) – At entry to study			Trail Making B (seconds) – At entry to Study		
Timing of Treatment	Treatment	n	Mean (Standard Deviation)	R^2^	Pr>F	Mean (Standard Deviation)	R^2^	Pr>F
After 24 hours	Placebo	9	38.4 (19.9)**	0.04	0.4243	62.2 (24.4)**	0.01	0.8941
After 24 hours	NAC	12	37.4 (20.7)**			70.3 (45.9)**		
Before 24 hours	Placebo	30	31.4 (11.3)**			66.5 (20.8)**		
Before 24 hours	NAC	27	32.4 (9.2)**			69.0 (21.7)**		

NAC administration restored normal TMT performance within 7 days after blast-induced mild traumatic brain injury (mTBI) (Table 7). The D7 MLR showed TMTA (F(1,74) = 6.64, p<0.05) and TMTB (F(1,74) = 4.866, p<0.05) scores were improved significantly by NAC treatment and scores for the NAC treated groups were equivalent to age-based norms. The D7 TMT scores for groups that did not receive NAC remained impaired. The D7 COWA and AN results showed no group differences (Tables 8 and 9).

**Table 7:** Neuropsychological tests: TMT at Day 7.

			Trail Making A (seconds) – Day 7			Trail Making B (seconds) – Day 7		
Timing of Treatment	Treatment	n	Mean (Standard Deviation)	R^2^	Pr>F	Mean (Standard Deviation)	R^2^	Pr>F
After 24 hours	Placebo	9	34.2 (18.3)**	0.09	0.0633	63.6 (16.3)**	0.07	0.1504
After 24 hours	NAC	12	23.4 (4.9)			50.2 (15.9)		
Before 24 hours	Placebo	30	27.0 (12.0)*			56.2 (20.1)*		
Before 24 hours	NAC	27	23.5 (7.7)			49.1 (17.1)		

* p<0.05 or **p<0.01 by z-test versus TMTA (23.7±7.8 seconds (S.D.)) or TMTB.

**Table 8:** Neuropsychological Tests: COWA and Animal Naming at Study Entry.

			COWA – At entry to study			Animal Naming – At entry to Study		
Timing of Treatment	Treatment	n	Mean (Standard Deviation)	R^2^	Pr>F	Mean (Standard Deviation)	R^2^	Pr>F
After 24 hours	Placebo	9	30.6 (8.0)	0.07	0.1245	17.4 (3.4)	0.02	0.7787
After 24 hours	NAC	12	35.3 (8.3)			19.7 (6.5)		
Before 24 hours	Placebo	30	39.3 (11.2)			19.4 (6.1)		
Before 24 hours	NAC	27	35.3 (10.1)			19.9 (6.9)		

**Table 9:** Neuropsychological Tests: COWA and Animal Naming at Day 7.

			COWA – Day 7			Animal Naming – Day 7		
Timing of Treatment	Treatment	n	Mean (Standard Deviation)	R^2^	Pr>F	Mean (Standard Deviation)	R^2^	Pr>F
After 24 hours	Placebo	9	35.6 (11.7)	0.03	0.5222	19.4 (7.7)	0.03	0.5994
After 24 hours	NAC	12	40.8 (9.4)			22.3 (6.3)		
Before 24 hours	Placebo	30	38.6 (15.6)			21.2 (6.0)		
Before 24 hours	NAC	27	42.3 (11.1)			22.5 (6.1)		

The D7 TMT performance paralleled the resolution of mTBI symptoms. Times were significantly shorter in symptom-free subjects (Bonferroni-adjusted t-tests, p<0.05). Subjects who were symptom free on D7 had normal TMTA and TMTB times, while the symptomatic subjects had prolonged times.

#### TM perforation and outcomes

TM perforation is a common combat-related blast injury [29], [30], [31], [32] that may indicate the magnitude of shock wave exposure [30], [33], [34] and be a proxy for blast over-pressure intensity [28], [29]. The number of symptoms and TMT test results at time of entry (day 0) were unaffected by TM status. TM perforation and NAC treatment status were both significant predictors of the number of day 3 symptoms (ANOVA, F(1,78) = 33.51, p<0.001 and F(1,78) = 4.64, p = 0.034, respectively). For the early treatment initiation group, the reduction of D7 symptoms by NAC treatment was independent of TM status (ANOVA, F (1, 56) = 12.19, p = 0.001). NAC treatment also produced significantly better resolution (no D7 symptoms) than placebo (logistic regression, p<0.005).

## Discussion

This is the first prospective, double-blinded, placebo-controlled randomized study to focus on the acute treatment of combat blast-related mTBI in a forward war zone. Supplementation of standard therapy with oral NAC had a significant impact on neuropsychological test results, number of mTBI symptoms, and complete symptom resolution by day seven of treatment when compared to placebo. Moreover, the pill form of NAC, the active ingredient in the FDA approved medication “Mucomyst”, produced no side effects in blast mTBI subjects. Although the study was powered only to examine the effects of NAC, there was a statistically significant secondary finding that standard treatment initiation within 24 hours had an independent benefit on neurological but not on neuropsychological outcome measures (TMT). A possible explanation for this difference is that the outcome measures assess the status of different neuronal circuitry components. The additive effects of NAC and early treatment produced 86% mTBI symptom resolution within seven days. Factors such as number of previous blast exposures, age and distance from the blast did not influence treatment outcomes significantly.

Headache and balance dysfunction are major clinical issues that arise acutely after blast exposure, impede return to duty, and can persist chronically [1], [4], [35]. Because they seriously impair performance in a combat environment, the initiation of standard treatment with oral NAC within 24 hours of mTBI is likely to have a definitive impact on battlefield end-strength and the readiness of troops in theater.

Performance on TMT neuropsychological tests was impacted significantly by blast mTBI and ameliorated by NAC administration. The initial test times were prolonged significantly at enrollment. NAC administration within 72 hours of injury produced normal D7 TMT times, but performance remained abnormal for subject groups that received only standard therapy. Because all subjects were tested on the same schedule and the reliability of repeated TRAIL making tests [25] is well documented, test-retest effects are not a confounding factor. Hence, TMTs are useful for documenting and monitoring cognitive status changes in acute blast mTBI.

The efficacy of NAC in early treatment of blast mTBI is consistent with its efficacy as a neuroprotective agent in ischemia-reperfusion cerebral stroke [6], [7], [8], closed head trauma [9], sensory nerve axotomy [10] and in the prevention of mitochondrial damage and loss of dendritic spines in hippocampal neurons [36] in animal models of closed head trauma and ischemia-reperfusion brain injury. A single, low level shock wave exposure to rodents can produce persistent biochemical changes in the hippocampus and cerebral cortex, accompanied by apoptotic cell death [37], [38], [39]. Even relatively low exposures produce very small parenchymal and subarachoid hemorrhages in 30–40% of exposed animals [40]. These findings suggest that vascular primary injury contributes to symptoms of mTBI, with slower development of neuronal damage [41]. Post-treatment with NAC has afforded protection against neuronal death in animal models. These neuroprotective effects reflect known antioxidant and anti-inflammatory effects [7], [8], [13], [14], [15], [36]. The cellular bases for memory and regulation of motivation properties within the nucleus accumbens may be improved by NAC activating neuronal cysteine-glutamate exchange and indirect effects on mGluR2/3 and mGluR5 [16] transmission. Finally, enhanced local bioavailability of NAC may be a natural consequence of vascular disruption in mTBI. Because NAC has limited capability to cross the normal blood-brain barrier [13], increased local brain permeability during vascular remodeling [42] may facilitate selective delivery to affected sites. A delayed opening of the blood-brain barrier from neuroinflammatory responses [42], [43], could create longer-term therapeutic opportunities.

Early symptomatic treatment initiation produced improvement that was statistically independent of effects of NAC treatment. The early and late subjects came from the same set of combat units with a shared history of living environment, combat exposure and similar clinical presentations. Although we believe that the time of enrollment was determined only by distance from TQ and availability of transport, it is impossible to know if there were unknown confounders between the two groups. However, we believe that the improvement seen in early subjects can be attributed to the concurrent standard symptomatic medical treatment and balance rehabilitation exercises begun earlier in the early treatment group. Exercise, in particular, can have neuroprotective pharmacomimetic effects on structures such as the hippocampus, possibly mediated by trophic factors [44].

Clinical trials in an active combat theater are subject to outside factors for “early termination” that do not arise in standard clinical environments. For this study, the opportunity for subject enrollment ended when combat operations terminated in this part of Iraq, prior to reaching the pre-determined number of enrollees for the trial. Because the available data were powered sufficiently to test the effects of NAC, we proceeded with analysis after all patients completed the protocol. The enthusiasm about the large treatment effect must be tempered by a recent review of the Cochran database [45], showing that studies with relatively small patient numbers and large odds ratios often show smaller odds ratios when the study is repeated. In this regard, we do note that the lower bounds of the 95% confidence intervals for early NAC treatment are reasonably large. It also should be noted that although we were able to draw some statistical conclusions the study was not powered to look at early vs. late treatment which argues for caution in interpreting that data.

One must be cautious to assert the therapeutic implications of NAC treatment for TBI within the limited scope of this study. While the results are very promising, the study was limited to evaluating a relatively small but representative sample of combat troops with acute mild head trauma and other minor injuries over one week of treatment. Some additional caveats are also inherent in the far-front battlefield environment. For example, study participants came from the same set of combat units with similar environmental exposure histories, living conditions (including the same forward operating bases), training, missions, and combat environments; combat personnel are predominantly males in their twenties. Therefore, it is prudent to consider several caveats for our findings. The study results do not imply any benefit for moderate or severe head trauma with significant surgical injuries. Because only one female was enrolled, the study may not generalize to all females. Although the study endpoint was only D7 there is some evidence to suggest that these effects may be long lasting. Tweedie, et al [46] have shown that mTBI triggers biochemical cascades within the first 24 hours which produce long term sequelae. Their work suggests that it is important to interrupt these cascades as early as possible. D7 resolution may indicate the lack of significant apoptotic and inflammatory changes in both grey and white matter. Nevertheless, the effects of treatment on longer term outcomes will need to be the subject of further study in a larger number of subjects.

The study can then, at least, be interpreted in a narrow fashion as showing a benefit of using NAC and early intervention for blast mTBI in an acute combat setting after mild blast exposure. The study brings up the intriguing possibility that NAC may be useful in other mTBI settings, but before this conclusion be reached much more study in the area and using this agent is required.

### Summary and Conclusions

We report the first double blinded placebo- controlled randomized study of a pharmaceutical countermeasure for the symptoms of blast-induced mTBI. All 81 subjects were seen within 72 hours of blast exposure by the same clinician-investigator at a forward location in an active combat zone. All medications and treatments were witnessed by a nurse, corpsman, or medic. The outcomes demonstrate that supplementation with oral NAC had a significant impact on neuropsychological test results, number of mTBI symptoms, and complete symptom resolution by day seven of treatment when compared to placebo. A secondary finding was that standard treatment initiation within 24 hours had an independent benefit on the neurological but not on neuropsychological outcome measures. Early treatment with NAC and standard therapy administered by a provider with expertise in mTBI care resulted in a seven day symptom resolution rate of 86% as compared to 11% in those receiving the same standard care by the same provider but who received placebo and began therapy between 24–72 hours after blast exposure. Additionally it should be noted that during this trial the pill form of NAC, the active ingredient in the FDA approved medication Mucomyst, produced no side effects in blast mTBI subjects. Mucomyst has an excellent safety profile in over forty years of use in hospitals worldwide. As such use of this medicine appears to be the first described pharmaceutical countermeasure for mTBI. These results while promising are still preliminary. This outcome needs to be supported by other studies of NAC for this pathophysiology examining neurosensory symptoms over a variety of time points both acute and chronic. Additionally, these findings argue for investigations of this therapy for other causes of traumatic brain injury.

## Supporting Information

Checklist S1 **CONSORT Checklist.**
(DOCX)

Protocol S1 **Trial Protocol.**
(DOCX)
