# Supplementary material for: Amelioration of Acute Sequelae of Blast Induced Mild Traumatic Brain Injury by N-Acetyl Cysteine: A Double-Blind, Placebo Controlled Study
Source: PLoS One. 2013 Jan 23;8(1):e54163. doi: 10.1371/journal.pone.0054163 (PMC3553161; doi:10.1371/journal.pone.0054163)
Supplement: Protocol S1 — Trial Protocol. (DOCX) [file pone.0054163.s002.docx]

**PROTOCOL TITLE: The use of anti-oxidants to reduce sequela of Mild TBI (mTBI) after blast exposure**

**PROTOCOL NUMBER: MNC-I-08-040**

**PRINCIPAL INVESTIGATOR:**

**____________________________ __________________________**

Michael E. Hoffer Date

CAPT MC USN

1^st^ Medical Battalion –Bravo Company

**MTF COMMANDER:**  I have reviewed this protocol and the investigator's required credentials including CITI completion and approve the protocol being forwarded for review.

**__________________________________ _____________________**

Phillip Sanchez Date

CDR MSC USN

1^st^ Medical Battalion –Bravo Company

**IRAQ RESEARCH DIRECTOR:** I have reviewed this protocol and recommend it being forwarded for additional review.

**___________________ _____________________**

Shad H. Deering Date

MAJ(P), MC

DC2RT Research Director

**MNC-I SURGEON**: I approve this protocol being forwarded for IRB review.

__________________________________ _____________________

Joseph Caravalho, JR Date

BG, MC

Surgeon, MNF-I and MNC-I

**USAISR SCIENTIFIC REVIEW**: Scientific review has been conducted on this protocol and is approved for forwarding to the IRB review.

__________________________________ _____________________

Charles E. Wade, PhD Date

Senior Scientist

**ASSURANCES:**

As the Primary Investigator on this protocol I acknowledge my responsibilities and provide assurances for the following:

**General Assurance:** I agree to conduct the study as outlined herein. I certify that all procedures involving human subjects have been described in full.

I agree that human subjects will be included in this study only after giving their free and informed voluntary consent. I will adhere to Army Regulation 70‑25 and AR 40-38 on the use of volunteers in research.

**Duplication of Effort:**  I have made a reasonable, good faith effort to ensure that this protocol is not an unnecessary duplication of previous experiments. I attest that MEDLINE AND DTIC literature searches have been performed and they indicate that the study is not an unnecessary duplication of effort.

**Statistical Assurance:** I assure that I have consulted with an individual who is qualified to evaluate the statistical design or strategy of this proposal, and that the "minimum number of subjects needed for scientific validity are used."

**Biohazard/Safety:** I have taken into consideration, and I have made the proper coordination regarding, all applicable rules and regulations regarding radiation protection, biosafety, recombinant issues, etc., in the preparation of this protocol.

**Training:** I verify that the personnel performing these procedures/manipulations described in this protocol are technically competent, have been properly trained, and are appropriately qualified.

**Compensation:** I am aware that I am not authorized to accept any form of personal compensation for my efforts in conducting this research.

**Modifications:** I am aware that any substantive modification to the protocol and the consent form must be approved by the BAMC Institutional Review Board (IRB) and, if applicable, to the Clinical Investigation Regulatory Office (CIRO) before implementation.

**Serious and Unexpected Adverse events:** I acknowledge that unanticipated problems involving risk to subject or others, serious and unexpected adverse experiences related to participation in the study and all subject deaths should be promptly reported in accordance with USAISR Policy and requirements of the Brooke Army Medical Center Institutional Review Board.

**Protocol Deviation:** I acknowledge that any protocol deviations discovered by either the PI or any auditing official will be immediately reported to the Chair, IRB. All corrective actions will be documented and become part of the master study file along with the report.

**Medical Monitor**: I am aware that a medical monitor must be assigned to greater than minimal risk protocols. The name and curriculum vitae of the medical monitor, who is someone other than the principal investigator, must be provided. This individual should be a qualified physician who is not associated with the protocol, and able to complete the requirements as outlined by the IRB.

**Publications**: I am aware that any presentation or publications resulting from this research must be cleared by the appropriate Public Affairs Office, undergo OPSEC review and be reviewed for release of actionable medical information.

**Data Sharing Agreement:** I am aware that data obtained from any of the USAISR databases such as JTTR, Burn Registry, Trauma Vitals, etc. will only be released to me based upon an IRB approved protocol. Such information will not be shared with anyone not authorized by the IRB to receive the information. Disposition of the data will occur as specified in the approved protocol. Any changes that impact on the type of data requested or in the utilization or disposition of the data will not occur until an amendment to the protocol is approved by the IRB. Upon completion of the study, all new data gained as a result of the study will be made available to the issuing database.

______________________________________ _______

Michael E. Hoffer, MC Date

CAPT, USN

**1.0 PROTOCOL TITLE:** The use of anti-oxidants to reduce sequela of Mild TBI (mTBI) after blast exposure

**2.0 PERSONNEL INVOLVED:**

**2.1 PRINCIPAL INVESTIGATOR:**

Michael E. Hoffer, MC

CAPT, USN

1^st^ Med Battalion, Bravo Company

TQ Surgical

Camp Al Taqaddum, Iraq

3422-710

Michael.hoffer@tq.mnf-wiraq.usmc.mil

**2.2 ASSOCIATE-INVESTIGATOR (S):** None

**2.3 TECHNICAL STAFF:** None

**2.4 ROLES AND RESPONSIBILITIES OF STUDY PERSONNEL:**

#### PRINCIPAL INVESTIGATOR: The principal investigator will carry out all aspects of the study including patient enrollment and consent, patient testing, data collection, and data analysis.

**ASSOCIATE INVESTIGATOR:** N/A

**TECHNICAL STAFF:** NA

**3.0 LOCATION OF STUDY:** USMC Level II Medical Facility – Al Taqeddum, Iraq

**4.0 TIME REQUIRED TOCOMPLETE STUDY:** 14 months

**Expected Start Date:** December 1, 2008

**Expected Completion Date:** February 1, 2010

**5.0 RESEARCH PLAN:** The goal of this study is to examine the use of one anti-oxidant, N acetylcysteine (NAC), in individuals who suffer blast injury and demonstrate early sequela of mTBI.

Recent changes in war fighting and operational situations have changed the injury patterns that our troops face in all aspects of military operations. Mild traumatic brain injury (mTBI) is among the most common injuries seen in the current theater of operations. While other injury patterns have shown a decrease over the last twelve months, mTBI from blast exposure has continued to be seen at increasing levels. This may be due in part to the fact that research has shown the individuals may suffer mTBI secondary to blast injury from one significant exposure or from repeated low level exposure which is more common as individuals rotate back to the theatre of operations for second, third, and even forth tours of duty. The sequela of mTBI include balance disorders, hearing loss, and cognitive dysfunction. These disorders can present immediately after the injury or several weeks after the injury but tests of function can detect injury shortly after the injury. Evidence exists in laboratory work and in human studies that anti-oxidant medicine can reduce/eliminate the sequela of mTBI if administered within 8-24 hours of injury.

**5.1 OBJECTIVES:**  A population and single-subject design double-blinded, placebo controlled study comparing the effectiveness of the observation and administration of the anti-oxidant NAC to administration of placebo and observation in individuals suffering the sequela of mTBI after a single or multiple blast exposures.

**5.2 HYPOTHESIS:** The administration of NAC for seven days along with observation will result in improved hearing and balance function in individuals who demonstrate these disorders after blast exposure when compared to the administration of placebo and observation at the seven day time point.

**5.3** **SPECIFIC AIMS:**

1. To determine the frequency of hearing loss and balance disorders in those exposed to blast injury that have non-emergent or non-urgent medical or surgical disorders and display a pattern of injury consistent with mTBI.

2. To determine the effectiveness of one week of anti-oxidant, N-acetylcysteine combined with observation, in the reduction of hearing loss and balance disorders one week after injury as compared to placebo medication and observation.

3. To determine the effectiveness of one week of anti-oxidant, N-acetylcysteine combined with observation, in the reduction of hearing loss and balance disorders six months after injury as compared to one week of placebo medication and observation.

**5.4 DESIGN:** This is a double-blind placebo controlled study that will take place at the level II treatment center at Al Taqaddum (TQ Surgical). The study will have two groups. One group of individuals who are treated with one week of N-acetylcysteine and observation and a second group (control) who will receive a placebo medicine and one week of observation. The aim of the study will be to initially compare the change/improvement in hearing function and balance function (as markers of mTBI symptomatology) at the one week post-injury point in the two groups. A secondary aim will be to examine the same functions at six-nine months after the date of injury which can occur when the primary investigator returns to CONUS.

Active duty service members who present to the clinic with complaints of dizziness, hearing loss, confusion, or any period of loss of consciousness after blast exposure will be the subjects of this study. Specific inclusion criteria and exclusion criteria (for example those requiring surgery) will be detailed below. Those eligible for participation in the study will be given informed consent (as detailed below) and those who agree to participate in the study will be randomized into the active medicine or placebo group (see below).

All individuals who agree to participate will undergo an “Otogram hearing test” and a dynamic gait index. (DGI) – methods explained below. Those in the medicine (NAC) treatment group will be given a loading dose of 8 grams (16– 500 mg tablets). For the next 72 hours, individuals will take 4.0 grams (10 tablets) divided into three doses each day**.** From day 4 –7 individuals will take 3 grams (6 tablets) divided into three doses a day. Individuals would also be observed taking these medicines for compliance. The placebo group will take the exact same number of tablets utilizing a placebo made by the company that makes the NAC which has been used without complication in previous Marine trials. I
Both groups of subjects will be maintained for observation for a seven day period of time (at the discretion of their commander). On the seventh day post-injury the individuals will be re-administered the hearing and balance tests.

Subject numbers will be given to each patient and the only identifiers kept will be date of birth, gender, distance from most recent blast, and number of blasts in the past. A key that ties a patient number to a specific individual will be kept under lock and key by the PI and utilized for the six-nine month after the date of injury follow-up. Those patients who return to Southern California will make up the longer term follow-up group. At some point during the six-nine month post-blast period these individuals will present to the balance and hearing clinic at Naval Medical Center San Diego (NMCSD). At NMCSD they will undergo a hearing test and a posturography balance evaluation as well as an Otogram test and a DGI. (all explained below).

A comparison of the group mean differences in hearing and balance function between tests on presentation day to day seven post-injury will provide the primary data analysis. A comparison of the group mean hearing function and group mean posturoraphy SOT score will be provide the secondary data analysis.

**5.4.1 Selection of subjects:** All active duty individuals who present to the level II surgical clinic at Al Taqeddum who meet the inclusion criteria and are not expressly excluded will be offered participation in the study. A total of seventy-five treatment and seventy-five control subjects will be included in the study. Only subjects who can give their own consent without dependence on surrogate consent will be offered enrollment in this study.

The principal investigator will determine if individuals meet inclusion criteria while the staff at TQ Surgical will determine if individuals are excluded from the study. In many cases this will happen de facto (patient taken to surgery, patient urgently medevac, etc.)

**5.4.1. a. Subject Inclusion:** Subjects meeting all of the following criteria may be enrolled in the study upon giving written informed consent:

1. Be active duty United States Service member

2. Be between the ages of 18 and 50 years

3. Present to TQ Surgical within 24 hours of blast exposure.

4. Have complains consistent with possible TBI including hearing loss, dizziness, lightheadedness, cognitive difficulties, and/or had loss of consciousness.

5. Are not expressly excluded by the criteria listed below.

**5.4.1. b. Subject Exclusion:** Subjects meeting any of the following criteria will be excluded from participation in the study:

1. Require urgent or emergent surgical procedures.

2. Will require medical evacuation to any level III or higher facility (other then to a level III for a CT scan only).

3. Has evidence of an open head wound.

4. Pregnant Females

5. The use, within the last seven days, of a nutritional supplement containing an anti-oxidant.

**5.4.1. c. Selection of control subjects:** The control group will only differ from the experimental group in that these individuals will get placebo medicine instead of active medicine. As the study is double blinded neither the subject nor the researcher will know what medicine the patient is taking.

**5.4.1. d. Control Subject Inclusion:** Same as above

**5.4.1. e. Control Subject Exclusion:** Same as above.

**5.4.1. f.** Pregnant women will be excluded from the study. A urine pregnancy test will be done prior to the start of the study to ensure female participants are not pregnant. The female subject will be instructed on the need to use a method of birth control or abstain from sexual relations during the study.

**5.4.1. g. Sample size:** The number of patients required for this study is 75 in each group. Because this is a novel study no pre-analysis for patient numbers required for significance (Power Analysis) can be provided. Due to the environment in which this study is to be carried out- the relevant data and statistics are not available. Patient numbers were determined based on estimated number of available patients.

**5.4.2 Measures:**

**5.4.2. a. Otogram hearing test:** The “Otogram” is a unique portable, self-administered hearing tests designed to give accurate audiometric tests in non-quiet, non-sound booth environments. The machine is composed of a touch screen display with simple instructions, insert ear phones for placement in the ear canals, a sound administration system capable of delivering the entire sound spectrum normally tested in standard audiograms, a bone vibrator, and a device that administers two spoken portions of the hearing test (the pure tone average and the word identification score) in any of six different languages. The device has been head to head tested with standard audiograms, in the audiology department of NMCSD, and has compared favorably in a wide range of patients. The device has been in use at level two medical facilities in Iraq for two years. The procedure requires fifteen minutes.

**5.4.2. b.** Dynamic Gait index: A highly reliable functional test of balance that results in an objective score. A copy of the DGI form is included as Appendix 1. The procedure can be completed in five minutes.

**5.4.2. c.** Standard audiometery (for six –nine months after the date of injury follow-up) – A comprehensive audiogram performed in a sound booth at a CONUS facility. Elements of this test include pure tone audiometry, bone and air conduction audiometery, word identification testing, acoustic reflex testing, and dynamic range testing. The procedure can be completed in fifteen minutes time.

**5.4.2. d.** Posturography (for six-nine month after the date of injury follow-up) – A device that is utilized at CONUS facilities. The device has a movable platform and a movable surround and tests the stability of a subject in a variety of situations (eyes closed, eyes open, surround stable, surround sway, platform stable, platform sway). After six conditions are tested a computerized sensory organization test (SOT) score is produced. The procedure requires ten minutes time.

**5.4.2.e**  Randomization. The medicine bags will be pre-randomized in conus and a key (identifying which bags contain which medicine will be kept under lock and key in the narcotic locker at TQ Surgical and in Dr. Hoffer’s office at NMCSD. Patients will be given medicine in bag order. First patient will get bag 1, second patient bag 2, etc. The number key from the bag will be placed into their medical record and in the data collection sheet in case the code needs to be broken.

**5.4.3 Data Collection:**  Each patient will provide initial hearing test (pure tone average and word identification scores) and balance test (DGI score) and will also provide these three pieces of data at seven days. A subset of patients will also provide a pure tone average, word identification score, and posturography SOT score at the 6-9 months after the date of injury. This subset is made up of individuals who are stationed in the San Diego/Camp Pendleton/29 Palms area. See data collection sheet.

**5.4.4. Departure from Protocol for Individual Subjects***:*

1. Patients wish to be discontinued from the study.

2. Patient condition requires medivac to higher level of care (except level III CT scans)

3. Patients cannot remain at TQ for seven days of observation.

**5.5 MILITARY RELEVANCE:** Mild traumatic brain injury is the most common injury seen in the current conflicts in Southwest Asia. It has been estimated that as many as 21% of those who deploy will suffer the sequela of mTBI. Among the most common sequela of mTBI are hearing loss and balance disorders both of which can be objectively measured in the field. Basic science work has demonstrated that short term and long term brain injury after blast can be dramatically reduced by giving an anti-oxidant before and/or after the blast exposure. This project represents a human trial of these experimental findings. It must be done in a situation where blast is occurring and where the medicine can be given within 24 hours of the blast exposure.

**5.6 MEDICAL APPLICATION:** The primary aim of this study is to examine the effect of the very early administration (1-24 hours after injury) of the anti-oxidant compound, NAC, on the short and long term sequela of mTBI. The study must be conduced in theatre to administer the medicine within 1-24 hours of injury and to examine short-term sequela.

**5.7 LITERATURE SEARCH:** Naval Medical Center San Diego, Library

**5.7.1. Literature Sources Searched:**  DTIC and MEDLINE.

**5.7.2. Date: September 2008**

**5.7.3. Key Words of Search:** Hearing loss, balance disorders, vestibular rehabilitation, anti-oxidants, and traumatic brain injury.

Current treatment of mTBI

Current US military treatment for mTBI consists of removal of a patient from the hazard and keeping the patient in a less hazardous environment (observation at their base of operations/off patrol) for at least seven days. Most of the data examining treatment strategies for mTBI has examined the hearing loss seen after impulse noise (acute acoustical trauma-AAT).

A variety of animal studies have looked at the efficacy of systemic corticosteroids as treatment for AAT. Generally some effect is seen, however, at very large mg/kg doses. Also, it seems that if too high a dose is used the corticosteroids treatment may actually be deleterious (d'Aldin et al., 1999, Lamm and Arnold, 1998). In addition, systemic corticosteroid use has the potential for a variety of serious or potentially serious side effects even with short term use including: psychosis, gastritis, hypertension, insulin resistance, sleep disturbances, and aseptic necrosis of the hip

Other treatments have been attempted clinically but there has been no convincing proof of efficacy of any of these treatments. In a retrospective study of 268 patients with AAT treated with nine different treatment regimens, no treatment regimen was found to be particularly effective compared to one another or untreated controls. These treatments included carbon dioxide inhalation, intravenous Dextran 40 with betahistine, pentoxifyllin, heparin or ginko-biloba added, or pentoxifyllin by itself, or Ringer’s solution plus ginko-biloba (Tschopp and Probst, 1989). A randomized, double blind, placebo-controlled study of dextran/pentoxifylline for acute acoustic trauma was reported (Probst et al., 1992). In this study no positive treatment effect was noted either. A study of the efficacy of hyperbaric oxygen (HBO) therapy for AAT was published. This study did report a benefit in hearing recovery for those receiving HBO therapy (Vavrina and Muller, 1995). However there is evidence from other investigators indicating that HBO may make AAT worse. Also, HBO therapy has potential serious side effects and it is not practical for far forward treatment of AAT. In a more recent study of war-related blast injury victims an intravenous infusion of vitamins plus pentoxifylline was found to be no more effective at reducing hearing loss than saline infusion (Sprem et al., 2001).

With regards to the other sequela of mild TBI (balance disorders, cognitive disorders, etc.) there has been no work demonstrating the efficacy of any early intervention as far as reducing the short term or long term effects of blast injury.

Rational for the study of N-acetylcysteine (NAC) as a treatment for mTBI

Histopathological damage to the ear by acoustic overexposure is often due to excessive oxidative stress and glutathione (GSH) depletion (Kopke et al., 1999, Kopke et al., 2000, Kopke et al., 2002). Acute acoustic over-stimulation is associated with the production of toxic free radical molecules and reactive oxygen species (ROS). This metabolic alteration may overwhelm the cochlea’s antioxidant defenses depleting the key cochlear endogenous antioxidant glutathione (GSH). This provides for the accumulation of ROS, which in turn leads to mitochondrial damage and eventually to the initiation of programmed cell death, also known as apoptosis. It is the accumulation of outer hair cell (OHC) death that contributes to permanent threshold shifts (PTS), and thus hearing loss (Hu et al., 2002). A similar pattern has now been demonstrated to occur after a blast pressure wave in cells throughout the brain particularly in the hair cells of the inner ear (hearing and balance cells).

The hypothesis established by our lab was that exogenous antioxidant replenishment of an intrinsic antioxidant glutathione (GSH) might prevent or treat the damaging effect of blast wave overpressure and thus reduce the damage seen after blast induced TBI. NAC is an effective agent for replenishing cellular GSH. Our lab and other investigators have conducted numerous experiments, using a variety of antioxidants, in a proven hearing loss animal model. The body of data that has been generated clearly supports the effectiveness of antioxidant therapy with NAC to attenuate the effects of acoustic over-stimulation leading to NIHL. NIHL is an effective marker for mTBI in animal models and the efficacy seen in reducing hearing loss should extend to other brain functions including balance and cognitive effects.

# Clinical Safety of NAC

NAC, a thiol containing amino acid derivative is used in the United States as a nutritional supplement. As it is devoid of serious side effects, and has proven potent antioxidant properties, it is a main ingredient in many over the counter nutraceutical preparations. However, unique to NAC, is it is also a drug which has the passed the stringent safety requirements for Food and Drug Administration (FDA) approval. Most of the abundant safety data for oral NAC is provided by its use in acetaminophen intoxication. In 1985, oral NAC emerged as the FDA-approved gold standard for safe and efficacious treatment/prevention of hepatic damage as a result of acute acetaminophen intoxication. NAC is effective for this indication as it replenishes liver GSH and scavenges ROS. A NAC dose of approximately 100 grams is administered orally over 72-hours and has proven to dramatically reduce the extent of liver injury, and yet has been associated with few side effects (Product Information, 1998). The most common side effects associated with this oral NAC regimen are gastrointestinal and dermatological in nature.

Safety of Oral N-acetylcysteine Administration

A plethora of safety experience has been amassed for the administration of oral NAC. NAC has been used in the clinical setting safely in countless patients for over 15 years. It is this experience in the general population that provides an adequate assessment of the risk, and therefore, confidence in the use of NAC for the treatment of AAT. Furthermore, the interest in this agent has also provided safety experience in numerous disease states and unique populations using similar treatment regimens to that proposed here for the treatment of AAT. The safety information from a small cross-section of these studies will be briefly discussed in support of the proposed NAC AAT treatment regimen. There are over 70 published placebo controlled studies on the use of NAC in the literature.

Wiklund and colleagues, in a randomized, double blind, crossover study, investigated the effect of NAC or placebo on homocysteine and lipoprotein (a) levels (Wiklund et al., 1996). Twelve hypercholesterolemic patients were treated with NAC doses of 2 grams twice daily for two weeks and placebo (with a week washout). Upon review of the tolerability and safety data, no difference was appreciated between the two treatment groups and it was concluded that NAC daily doses of 4 grams were well tolerated. Furthermore, no changes from baseline were observed in the clinical laboratories regardless of treatment. Two complaints of flatulence and bad taste were reported for the subjects receiving NAC. A single incidence of flatulence and bad taste was reported when subjects received the placebo regimen, these subjects had also reported 1 of the 2 events experienced when receiving NAC (Wiklund et al., 1996). These findings were obtained in a population without significant disease and in a dosing regimen most similar to that proposed for this study. Therefore, these data are most compelling when assessing the potential safety of NAC administration for an AAT indication.

In a recent randomized, double blind, placebo controlled study conducted in a Marine’s in a training environment who were given NAC or placebo medicine three times a day during a ten day rifle training evolution the side effect profile from NAC was below that of placebo and in both cases consisted of a mild stomach upset which was no higher than that seen in Marines in this training evolution not receiving medicines (Balough, et al)

### Clinical Safety Experience with Oral NAC in Acetaminophen Intoxication

The most abundant safety data for oral NAC administration is provided by its use in acetaminophen intoxication. In the 1985, short duration, high oral dose NAC emerged as the FDA approved gold standard for safe and efficacious treatment/prevention of hepatic damage as a result of acute acetaminophen intoxication. Acetaminophen overdoses greater than 150 mg/kg are hepatotoxic and commonly result in a rapid and permanent hepatocellular necrosis related to oxidative stress and GSH depletion that often leads to hepatic failure and death. Oral NAC is administered for this indication at a loading dose of 140 mg/kg (¡Ö 11 grams) followed by a maintenance dose of 70mg/kg (¡Ö 5.5 grams) given every four hours for a total of 72 hours. This regimen has proven to dramatically reduce the extent of liver injury when administered within 24 hours of overdose. Most interesting, few side effects have been associated this regimen despite a large total NAC dose (¡Ö100 grams) administered over just 72-hours (Product Information, 1998). The most common side effects associated with this short duration, high dose oral NAC regimens are gastrointestinal and dermatological. Specifically, nausea, vomiting, and diarrhea are reported with the oral NAC acetaminophen overdose regimen. With rare occurrence, dermatological events of rash and urticaria have also been reported American Society of Health-Systems Pharmacists (2000). However, it is difficult to determine the frequency of these events as they are confounded by the underlining acetaminophen overdose, which also causes these gastrointestinal symptoms. One large study investigated 1283 patients receiving the NAC regimen and concluded that no event, other than GI, was reported with a frequency greater than 5 percent (Miller and Rumack, 1983). The only serious event reported in the literature in over 15 years of clinical experience with this oral NAC regimen was a single incidence of angioedema in a 25-year old man. A patient receiving the standard NAC regimen for acetaminophen overdose administration developed a rash and swelling of the tongue. Diphenhydramine and methylprednisolone were administered, eliminating the angioedema, allowing the NAC regimen to be completed.

Novel Contributions of this Study

This study seeks to answer an important question that has not been investigated in the past in a theatre environment. The hypothesis is that the acute damage seen after blast injury is reversible in the short term if the appropriate medicine is given within a critical time period. Based on laboratory work we have reason to believe that anti-oxidants may be an ideal medicine for this task and have demonstrated this effect in a noise induced hearing loss model in Marines in a training environment. However, our work indicates that there is a very short time window in which the damage done may be reversed (or further damage prevented). This hypothesis has not been tested for the sequela of mTBI as no “model setting” outside of the current operational theatre can adequately reproduce the effects seen in theatre. This study would be the first study examining acute anti-oxidant administration for the treatment mTBI in a real operative setting where blast is a predominant injury vehicle.

**6.0 HUMAN SUBJECT PROTECTION**

**6.1 NATURE AND LOCATION OF DATABASE(S):**  Each measure will have a numeric number linked to the subject name. When transferring the data from the measures into an Excel database, only the numeric number, and not the subject’s name, will be used. Thereafter, the hard copies of the measures will be stored in a subject file, which, in turn, will be stored in a locked file cabinet in the office of the PI. No person outside the study will have access to any study information than can be identified with any subject.

**6.2 RECORDING OF EXTRACTED DATA WITH IDENTIFIERS:** The direct identifier is date of birth. Each subject will also be given a coded, randomly assigned number identifier.

**6.3 LOCATION OF EXTRACTED AND RECORDED DATA:** All data will be identified with a study participant number and stored on-site electronically on a secure, password-protected and encrypted computer. Any computer used for storing data will not at anytime be connected to the server. All data will be kept in the PI's files until the study is complete. Data with only a coded identifier will be encrypted and password protected. Only the PI will have access to any files that include subject identifying information. The data which is collected in this study will be kept as confidential as possible.

**6.4 TRANSMISSION OF EXTRACTED DATA FOR COLLABORATIVE RESEARCH: N/A**

**6.5 LINKAGE OF EXTRACTED DATA TO OTHER DATABASES:** **N/A**

**6.6 STATUS OF THE EXTRACTED DATA AFTER COMPLETION OF THE RESEARCH STUDY:** Group data will be summarized in final report documents; however, subjects’ names will never be published as a contributor of specific data points or as a participant in the investigation. Once the study is completed and the published reports are approved, the subject files will be destroyed.

When the results of the research are published or discussed in conferences, no information will be included that would reveal the subject's identity. Records of the subject's participation in this study may only be disclosed in accordance with federal law, including the Federal Privacy Act, 5 U.S.C. 552a, and its implementing regulations. DD Form 2005, Privacy Act Statement-Health Care Records, contains the Privacy Act Statement for the records. By signing the consent form document, the subject gives permission for information gained from participation in this study to be published in medical literature, discussed for educational purposes, and used generally to further medical science. The subject will not be personally identified; all information will be presented as anonymous data.

**6.7 BENEFITS:** Those individuals receiving the study medicine have the potential benefit of a reduction of the sequela of TBI as the mechanism of action of the medicine suggests that this result may occur. However we cannot assure any participant of any particular benefit.

**6.8 RISKS:** All patients entered into the study will receive at least current standard of care treatment. The tests being performed (hearing and balance tests) present no risk to the patients. The medicine group of patients is taking a dose of anti-oxidant (NAC) which has already been shown in a very large Marine recruit trial to have no significant risk. In this study the adverse events from NAC (stomach upset) were identical to placebo and non-drug levels. There is a long history of NAC use (see background section) with extensive documentation of the safety of this medicine.

**6.8.1. Adverse Events:**

Unanticipated problems involving risk to subjects or others, serious adverse events related to participation in the study and all subject deaths should be promptly reported, through the USAISR Regulatory Compliance Office. A complete written report should follow the initial telephone call or the e-mail.

All other adverse events will be reported to the IRB during continuing review time.

**6.8.2. Medical Care for Research Related Injury:** If volunteers are injured as a result of taking part in this study, medical care for the research related injured will be provided at no additional cost to the subject. This is not a significant issue as all participants will be active duty patients.

**6.8.3. Biohazards/Safety:** None

**7.0 DATA ANALYSIS PLAN:**  An intent-to-treat methodology will be utilized for this study. Statistical significance will be defined as an alpha level of 0.05. No interim analyses of efficacy data are planned. All relevant study data will be summarized. For continuous and pseudo-continuous data the number of observations, mean, standard deviation, median, 1st quartile, and 3rd quartile will be computed. For binary and discrete data the number of cases, number of observations, and percentage will be computed. Other methods such as graphic approaches may be used if deemed necessary**.**

**8.0 BIBLIOGRAPHY:**

Attias, J., Weisz, G., Almog, S., Shahar, A., Wiener, M., Joachims, Z., Netzer, A., Ising, H., Rebentisch, E. and Guenther, T. (1994) Oral magnesium intake reduces permanent hearing loss induced by noise exposure. *Am. J. Otolaryngol.,* **15,** 26-32.

Balough BJ, Bauman, N. and Jastreboff, P. F. (1999) In *Proceedings of the Sixth International Tinnitus Seminar*(Ed, Hazell, J.) London, Tinnitus and Hyperacusis Centre, pp. 403-406.

d'Aldin, C., Cherny, L., Devriere, F. and Dancer, A. (1999) Treatment of acoustic trauma. *Ann N*

*Y Acad Sci,* **884,** 328-44.

De Rosa, S. C., Zaretsky, M. D., Dubs, J. G., Roederer, M., Anderson, M., Green, A., Mitra, D., Watanabe, N., Nakamura, H., Tjioe, I., Deresinski, S. C., Moore, W. A., Ela, S. W., Parks, D. and Herzenberg, L. A. (2000) N-acetylcysteine replenishes glutathione in HIV infection. *Eur J Clin Invest,* **30,** 915-29

.

Duan, M., Qiu, J., Olofsson, A., Borg, E. G. and Laurell, G. (2002) In *Association for Research in Otolaryngology*St. Pete's Beach, FL.

Flottorp, G. (1991) Treatment of noise induced hearing loss. *Scand Audiol Suppl,* **34,** 123-30.

Hoffer ME, Gottshall KR, Moore RJ, Balough BJ, Wester DC. Characterizing and Treating Dizziness after Mild Head Trauma. Otol/Neurotol, 25(2);135-38, 2004.

Hoffer ME, Gottshall KR, Moore RJ, Wester D. Characterizing Dizziness after Head Trauma**.** Presented at the XXIII Barany Society Meeting, Paris, France, July 7-9, 2004

Hu, B. H., Henderson, D. and Nicotera, T. M. (2002) Involvement of apoptosis in progression of cochlear lesion following exposure to intense noise (1). *Hear Res,* **166,** 62-71.

Kopke, R. D., Allen, K. A., Henderson, D., Hoffer, M., Frenz, D. and Van De Water, T. (1999) *A radical Demise: Toxins and trauma share common pathways in hair cell death,* Annals of the New York Academy of Sciences, New York.

Kopke, R. D., Coleman, J. K., Liu, J., Campbell, K. C. and Riffenburgh, R. H. (2002

Enhancing Intrinsic Cochlear Stress Defenses to Reduce Noise-Induced Hearing Loss. *Laryngoscope. 112(9):1515-1532, September 2002*

Kopke, R. D., Coleman, J. K., Liu, J., Campbell, K. C. and Riffenburgh, R. H. (2002) Candidate's thesis: enhancing intrinsic cochlear stress defenses to reduce noise-induced hearing loss. *Laryngoscope,* **112,** 1515-32.

Kopke, R. D., Jackson, R. L., Henderson, D., Liu, J. and Coleman, J. K. M. (2003) In *Association for Research in Otolaryngology* Daytona, FL.

Kopke, R. D., Weisskopf, P. A., Boone, J. L., Jackson, R. L., Wester, D. C., Hoffer, M. E., Lambert, D. C., Charon, C. C., Ding, D. L. and McBride, D. (2000) Reduction of noise-induced hearing loss using L-NAC and salicylate in the chinchilla. *Hear Res,* **149,** 138-46.

Lamm, K. and Arnold, W. (1998) The effect of prednisolone and non-steroidal anti-inflammatory agents on the normal and noise-damaged guinea pig inner ear. *Hear Res,* **115,** 149-61.

Levine, R. A. (1999) In *Proceedings of the Sixth International Tinnitus Seminar*(Ed, Hazell, J.) London, Tinnitus and Hyperacusis Centre, pp. 193-197.

Meikle, M. B. (1992) In *Tinnitus 91. Proc. IV Internat. Tinnitus Seminar*(Ed, Aran, J.-M.) Kugler, Amsterdam, pp. 555-62. Miller, L. F. and Rumack, B. H. (1983) Clinical safety of high oral doses of acetylcysteine. *Semin Oncol,* **10,** 76-85.

Newman, C. W., Jacobson, G. P. and Spitzer, J. B. (1996) Development of the Tinnitus Handicap

Inventory. *Arch Otolaryngol Head Neck Surg,* **122,** 143-8. Ohinata, Y.,

Miller, J. M. and Schacht, J. (2003) Protection from noise-induced lipid peroxidation and hair cell loss in the cochlea. *Brain Res,* **966,** 265-73.

Pfander, F., Bongartz, H., Brinkmann, H. and Kietz, H. (1980) Danger of auditory impairment from impulse noise: A comparative study of the CHABA damage-risk criteria and those of the Federal Republic of Germany. *J Acoust Soc Am,* **67,** 628-33.

Probst, R., Tschopp, K., Ludin, E., Kellerhals, B., Podvinec, M. and Pfaltz, C. R. (1992) A randomized, double-blind, placebo-controlled study of dextran/pentoxifylline medication in acute acoustic trauma and sudden hearing loss. *Acta Otolaryngol,* **112,** 435-43.

Rabinowitz, P. M., Pierce Wise, J., Sr., Hur Mobo, B., Antonucci, P. G., Powell, C. and Slade, M. (2002) Antioxidant status and hearing function in noise-exposed workers. *Hear Res,* **173,** 164-71.

Royster, J. D. and Royster, L. H. (1986) Using audiometric data base analysis. *J Occup Med,* **28,** 1055- 68.

Sprem, N., Branica, S. and Dawidowsky, K. (2001) Vasodilator and vitamins in therapy of sensorineural hearing loss following war-related blast injury: retrospective study. *Croat Med J,* **42,** 646-9.

Taggart, R. T., Wolgemuth, K. S., Williams, E. A., Smith, S., Marshall, L. and Kopke, R. D. (2001) In *Twenty-first Midwinter Research Meeting of the Association for Research in Otolaryngology*Association for Research in Otolaryngology, St. Petersburg Beach, FL.

Tschopp, K. and Probst, R. (1989) Acute acoustic trauma. A retrospective study of influencing factors and different therapies in 268 patients. *Acta Otolaryngol,* **108,** 378-84.

Vavrina, J. and Muller, W. (1995) Therapeutic effect of hyperbaric oxygenation in acute acoustic trauma. *Rev Laryngol Otol Rhinol (Bord),* **116,** 377-80.

Wiklund, O., Fager, G., Andersson, A., Lundstam, U., Masson, P. and Hultberg, B. (1996) N-acetylcysteine treatment lowers plasma homocysteine but not serum lipoprotein(a) levels. *Atherosclerosis,* **119,** 99-106.

**9.0 BUDGET:** Funds will be required to conduct the study (buy the medicine) but these funds already exist as this study is pre-funded by Office of Naval Research.

### 10.0 SAMPLE DATA COLLECTION SHEET/CASE REPORTING FORMS

| Subject # | DOB | Med bag # | Sex (M/F) | Blast # | Distance | PTA (i) | Discrimination (i) | DGI (i) |
| --- | --- | --- | --- | --- | --- | --- | --- | --- |
|  |  |  |  |  |  | PTA (3) | Discrimination (3) | DGI (3) |
|  |  |  |  |  |  | *PTA (7)* | *Discrimination (7)* | *DGI (7)* |
|  |  |  |  |  |  | OPTA (6m) | ODiscrimination (6m) | DGI (6 mo) |
|  |  |  |  |  |  | APTA (6m) | ADiscriminaion (6 m) | SOT (6mo) |
|  |  |  |  |  |  |  |  |  |
|  |  |  |  |  |  |  |  |  |
| 1. XXXX |  |  |  |  |  |  |  |  |
|  |  |  |  |  |  |  |  |  |
|  |  |  |  |  |  |  |  |  |
|  |  |  |  |  |  |  |  |  |
|  |  |  |  |  |  |  |  |  |
|  |  |  |  |  |  |  |  |  |
| 2.XXXX |  |  |  |  |  |  |  |  |
|  |  |  |  |  |  |  |  |  |
|  |  |  |  |  |  |  |  |  |
|  |  |  |  |  |  |  |  |  |
|  |  |  |  |  |  |  |  |  |
| 3.XXXX |  |  |  |  |  |  |  |  |
|  |  |  |  |  |  |  |  |  |

Sample DGI

| WALK AT NORMAL SPEED |  |
| --- | --- |
| CHANGE IN GAIT SPEED |  |
| GAIT WITH HORIZONTAL HEAD MOTION |  |
| GAIT WITH VERTICAL HEAD MOTION |  |
| GAIT AND PIVOT TURN |  |
| STEP OVER OBSTACLE |  |
| STEP AROUND OBSTACLE |  |
| WALK UP STAIRS |  |
| TOTAL |  |

##### 11.0 IMPACT STATEMENT

**PROTOCOL TITLE/NUMBER: The use of anti-oxidants to reduce sequela of Mild TBI (mTBI) after blast exposure**

**PRINCIPAL INVESTIGATOR: Michael E. Hoffer, CAPT MC USN**

**LOCATION OF STUDY:** *Al Taqeddum, Iraq*

**ASSISTANCE REQUESTED**: None

**TOTAL NUMBER OF PATIENTS/SUBJECTS** **TO BE STUDIED:** 150

**LENGTH OF STUDY: 14 months**

**NUMBER OF SAMPLES/ACTIVITIES PER MONTH**: 24

**FUNDING REQUIREMENT**: Pre-funded by Office of Naval Research

X Approved, no comment.

 Approved with comment.

 Disapproved cannot support activity.

Michael E. Hoffer, MC

CAPT, USN

Chief, Otolaryngology, TQ Surgical
